# Expanding the Microbial Genomic Landscape and Biotechnological Applications of CRISPR-Cas Systems

**DOI:** 10.3390/biology15100748

**Published:** 2026-05-08

**Authors:** Swati Singh, Harshita Tiwari, Mamta Singh, Vibhav Gautam, Anju Gautam, Hemant Kumar Gautam

**Affiliations:** 1 Centre of Experimental Medicine and Surgery, Institute of Medical Sciences, Banaras Hindu University, Varanasi 221005, India; swati.7672@bhu.ac.in (S.S.); harshitatiwari@bhu.ac.in (H.T.); anjugautam151@gmail.com (A.G.); 2 Department of Obstetrics and Gynaecology, Institute of Medical Sciences, Banaras Hindu University, Varanasi 221005, India; mamta.singh1@bhu.ac.in; 3 School of Biosciences, University of Kent, Canterbury CT2 7NZ, UK; 4 Department of Biotechnology and Bioinformatics, Delhi Skill and Entrepreneurship University, Dwarka, New Delhi 110077, India

**Keywords:** CRISPR-Cas, PAM, epigenetic editing, Cas9, guide RNA

## Abstract

The CRISPR-Cas systems, identified initially as adaptive immune mechanisms in bacteria and archaea against viral threats, have rapidly evolved into transformative tools in genetic engineering and biotechnology. These RNA-guided systems are broadly classified into Class 1, comprising multi-subunit complexes, and Class 2, characterized by compact single-effector protein, such as Cas9, Cas12, and Cas13. Their remarkable structural and functional diversity enables microorganisms to adapt to diverse ecological niches, offering a vast repertoire of genome-editing strategies. Beyond their natural role in maintaining genome integrity and defense, CRISPR-Cas systems have been extensively repurposed for precise genome modification, transcriptional regulation, epigenetic editing, and nucleic acid detection. Recent advances in computational mining of microbial genomes and metagenomes have uncovered a broad range of novel CRISPR effectors with unique properties, distinct protospacer adjacent motif (PAM) requirements, RNA-targeting capabilities, miniature architectures, and promiscuous cleavage activities that significantly expand the molecular biology toolkit. The development of CRISPR-based technologies such as base editing, prime editing, gene knock-in/out, and live-cell DNA/RNA imaging exemplifies the versatility of these systems. Despite the challenges associated with delivering complex Class 1 systems, both classes are now being actively harnessed across diverse microbial platforms. Concurrently, the CRISPR-Cas research, particularly for guide RNA (gRNA) design and activity prediction, has revolutionized target specificity and editing efficiency. This review presents a comprehensive overview of CRISPR-Cas system diversity, their genomic landscape in microorganisms, and their cutting-edge biotechnological applications. It also emphasizes the transformative potential of CRISPR in synthetic biology, therapeutics, diagnostics, environmental remediation, and agriculture, while also addressing the ethical and biosafety considerations surrounding its deployment. As CRISPR-Cas systems continue to evolve, they stand at the forefront of innovations that bridge natural microbial immunity with engineered precision tools for next-generation biotechnology.

## Simple Summary

A natural defense system has been discovered by scientists in bacteria that helps them defend against viruses. This system, known as clustered regularly interspaced short palindromic repeats and associated proteins, has become an important biological tool in modern medicine. It allows researchers to make precise changes to DNA, the genetic material that carries instructions for life. Over time, scientists have identified many different types of this system in various microorganisms, ranging from simple to more complex forms. These natural variations have provided researchers with a wide range of tools that can be used to detect diseases, improve crops, study genes, and develop new medical treatments. Recent advancements in computer-based analysis and DNA sequencing have enabled scientists to discover new versions of gene-editing proteins with improved features. Some change specific parts of DNA, while others detect genetic material associated with infections or diseases. These discoveries have greatly expanded the possibilities for research and practical applications. Although this technology holds enormous promise in medicine, agriculture, and environmental protection, it is important to use it effectively and judiciously. Careful attention to safety and ethical concerns will further benefit society in a responsible and sustainable way.

## 1. Introduction

The ability to precisely manipulate genetic information has revolutionized modern biology and biotechnology, enabling remarkable precision and control over cellular functions and biological systems. Targeted genome-editing technologies allow researchers to investigate gene function, regulate complex genetic networks, and engineer organisms with desirable traits. Among these technologies, Clustered Regularly Interspaced Short Palindromic Repeats (CRISPR)-Cas systems have emerged as one of the most transformative innovations in molecular biology, offering a simple, efficient, and programmable platform for targeted genome modification across diverse organisms [1]. The CRISPR-Cas system was first identified in 1987 during a study on the *Escherichia coli* genome, where unusual repeat sequences were observed while studying genes involved in alkaline phosphatase isozyme conversion [2]. A similar sequence was later reported in archaeal genomes such as *Haloferax mediterranei* and subsequently across various bacterial and archaeal genomes [3]. In 2002, Ruud Jansen and colleagues coined the term CRISPR and identified the associated CRISPR-associated (*Cas*) genes, establishing the basis for understanding this prokaryotic defense mechanism [4]. Subsequent studies revealed that CRISPR-Cas systems function as heritable defense mechanisms, incorporating fragments of invading viral or plasmid DNA as spacer sequences within CRISPR arrays, thereby enabling sequence-specific recognition and elimination of foreign genetic material during subsequent infections [5].

CRISPR and its associated Cas proteins form a sophisticated and adaptive immune system found in approximately half of the bacterial species and nearly all archaea. These systems protect microbes from invading nucleic acids, such as bacteriophage genomes and plasmids, through a sequence-specific mechanism that is both programmable and inheritable [6]. Genomically, CRISPR-Cas systems are typically organized into two main components: a CRISPR array and an adjacent *Cas* gene operon. The CRISPR array comprises short direct repeats interspaced with unique spacer sequences derived from previously encountered foreign genetic elements, functioning as a molecular memory of past infections. The associated *Cas* genes encode proteins that operate through the tripartite mechanism, i.e., adaptation, CRISPR RNA (crRNA) biogenesis, and interference. During adaptation, segments of invading genetic material (protospacers) are incorporated into the CRISPR array as new spacers. In some CRISPR systems, an associated reverse transcriptase (RT) domain is also associated with the adaptation machinery and facilitates the acquisition of spacers derived from RNA templates. During the crRNA processing phase, the array is transcribed into a precursor crRNA (pre-crRNA), which is subsequently processed into mature crRNAs. In the final interference stage, these crRNAs guide Cas effectors to recognize and degrade complementary sequences in foreign DNA or RNA [7]. CRISPR-Cas systems exhibit substantial diversity and are broadly classified into two major classes based on the architecture of their effector complexes, which are Class 1 and Class 2 systems. Among Class 2 CRISPR-Cas systems, the Type II system, particularly the *Streptococcus pyogenes* Cas9 (SpCas9), has become the most widely used genome-editing tool. In this system, a single-guide RNA (sgRNA) directs Cas9 to a complementary DNA sequence adjacent to a PAM, where the nuclease introduces a site-specific double-strand break (DSB) that can subsequently be repaired through cellular DNA repair pathways [8,9].

Beyond genome editing, CRISPR technologies have enabled the development of several powerful derivative platforms, including prime editors (PEs), base editors (BEs), CRISPR activation (CRISPRa), and CRISPR interference (CRISPRi). In addition, naturally occurring CRISPR-associated transposases (CASTs) represent a distinct class of CRISPR-linked systems that enable RNA-guided DNA integration [10,11,12]. More broadly, the performance metrics of CRISPR-based systems such as editing efficiency, PAM constraints, and delivery feasibility are strongly influenced by the host organism. While many reported values are derived from model organisms such as *E. coli* and *B. subtilis*, these parameters may differ considerably in non-model microbial systems due to variations in transformation efficiency, DNA repair mechanisms, and cellular physiology. Notably, while some platforms such as BEs can achieve very high editing efficiencies (>80–90%), they typically reflect performance at compatible target sites within the defined editing windows and for transition mutations. The mechanistic constraints primarily limit the range of editable loci rather than efficiency at permissible loci. Recent advances in CRISPR technology have led to the development of diverse genome-editing platforms with distinct mechanisms, efficiencies, and applications in microbial systems. A comparative overview of these advanced CRISPR tools is summarized in Table 1.

CRISPR technologies have also facilitated the engineering of microorganisms for industrial and biomedical applications. The CRISPR-Cas9 system is a programmable, targeted endonuclease that promotes precise modification of endogenous genomic loci that facilitates the systematic investigation of genetic variations and functional genomic elements. Engineered microbial chassis strains are increasingly used as efficient cell factories for the sustainable production of valuable biomolecules, while gene-edited probiotics show enhanced therapeutic and nutritional functions. Additionally, catalytically inactive Cas9 (dCas9) has been repurposed as a programmable platform for gene regulation, enabling targeted transcriptional repression or activation through fusion with regulatory domains [13,14]. Despite its widespread application, Cas9 presents several limitations, including its relatively large size (~1300 amino acids), the constraint of PAM sequence requirements, and off-target effects that compromise editing precision. The large size of CRISPR nucleases such as Cas9 and Cas12a can hinder efficient packaging and delivery into cells, particularly when using size-limited viral vectors such as adeno-associated viruses (AAVs) that are widely employed for in vivo gene therapy. Furthermore, stringent PAM sequence requirements restrict the number of accessible genomic target sites, thereby limiting editing flexibility in certain regions of the genome. These limitations have motivated the exploration of compact Cas orthologs and engineered CRISPR variants with improved delivery compatibility, relaxed PAM recognition, and enhanced specificity for genome-editing applications. Among these, the compact Cas9 ortholog derived from *Neisseria meningitidis* (Nme2Cas9) has emerged as a promising candidate due to its smaller size, compatibility with single-AAV delivery systems, and high genome-editing specificity [15]. CRISPR-Cas9 technology has demonstrated remarkable versatility across multiple fields. In biomedical research, it has advanced gene function analysis, disease modeling, and the development of therapeutic strategies. For example, Kim et al. utilized CRISPR-Cas9 for the understanding of disease-associated mutational variants, which enables editing of multiple genes simultaneously, highlighting its power in functional genomics [16]. Beyond medicine, CRISPR-Cas9 is also transforming agriculture by enabling precise genetic modifications to improve crop traits such as disease resistance, yield, and nutrition. Akama et al. applied this approach to enhance the nutritional quality of grains by targeting the *OsGAD3* gene that further leads to high GABA content, highlighting the potential of CRISPR technologies for crop biofortification [17]. Overall, CRISPR-Cas9 offers a precise, efficient alternative to traditional methods in both science and industry. Despite these advances, significant challenges remain in identifying novel CRISPR systems with improved efficiency, compact size, and broader targeting capabilities. Recent advances in genome and metagenome mining have revealed an expanding diversity of CRISPR-Cas systems, offering new opportunities to develop next-generation genome-editing tools.

In this review, we summarize the diversity and classification of CRISPR-Cas systems, highlight recent advances in CRISPR-based genome-editing technologies, and discuss emerging strategies for discovering novel CRISPR effectors through genomic and metagenomic mining approaches. We further examine the applications of these systems in microbial engineering, biotechnology, and therapeutic development, and outline current challenges and future perspectives in the rapidly evolving field of CRISPR-based genome editing.

**Table 1:** Comparative overview of advanced CRISPR-based genome-editing platforms used in microbial systems.

Parameter	CRISPR Interference (CRISPRi)	CRISPR Activation (CRISPRa)	Base Editors (BEs)	Prime Editors (PEs)	CRISPR- Associated Transposases (CASTs)
**Core** **mechanism**	Blocks transcription of the target gene by dCas9-sgRNA complex binding to the promoter or coding region	Fusion of dCas9 with transcriptional activators to enhance gene expression	Fusion of Cas9 nickase/dCas9 with cytidine or adenine deaminase	Fusion of Cas9 nickase with reverse transcriptase guided by prime editing guide RNA (pegRNA)	RNA-guided transposase complex integrates DNA at target sites
**Type of** **genetic** **modification**	Gene repression (transcriptional silencing)	Targeted gene activation	Single nucleotide transitions (C→T, A→G)	All base substitutions, small insertions and deletions	Programmable integration of large DNA fragments
**Editing** **efficiency**	Moderate to high depending on sgRNA position	Moderate and depends on promoter architecture	Very high (often >80–90% editing in many bacteria)	Moderate and typically lower than BEs	Variable and generally lower than BEs but suitable for large DNA integration
**Editing scope**	Regulation of gene expression	Programmable gene activation	Precise point mutations and gene inactivation	Precise genome rewriting including substitutions, insertions and deletions	Enables kilobase-scale DNA insertions without DSBs
**PAM requirements**	Strict (SpCas9 NGG PAM sequence commonly used)	Strict (SpCas9 NGG PAM sequence commonly used)	PAM-dependent targeting; engineered Cas variants expand scope	PAM-dependent targeting; more restrictive due to pegRNA constraints	PAM-dependent; limited targeting range
**Payload size**	Low (dCas9 + sgRNA)	Moderate (dCas9 + activator fusion)	Moderate (Cas9-deaminase fusion)	High (Cas9-RT fusion + pegRNA)	Very high (multi-protein complexes, >10 kb)
**Off-target effects**	Low (no DNA cleavage)	Low–moderate (transcriptional noise)	Moderate (bystander edits within editing window)	Lower than BEs but still under investigation	Low insertion specificity in some systems
**Typical** **microbial** **applications**	Functional genomics, metabolic pathway modulation	Metabolic engineering and synthetic gene circuits	Metabolic engineering, strain improvement, antibiotic resistance studies	Precision genome engineering, regulatory element modification	Synthetic pathway integration, horizontal gene transfer (HGT) studies
**Advantages**	Reversible, no DNA cleavage	Programmable transcriptional control	High efficiency, no DSBs, minimal cytotoxicity	Highly versatile editing capabilities	Enables large DNA insertions without homologous recombination
**Mechanistic trade-offs**	Repression efficiency depends on sgRNA placement; possible partial gene silencing	Activation varies with promoter accessibility; overexpression may cause metabolic burden	Limited to transition mutations and editing window constraints	Complex pegRNA design and relatively lower efficiency	Large multi-protein complexes reduce targeting efficiency; limited precision for single-base edits
**References**	[18,19,20,21]	[22,23]	[24,25,26]	[27,28,29]	[30,31,32]

## 2. Diversity and Classification

**Figure 1:**
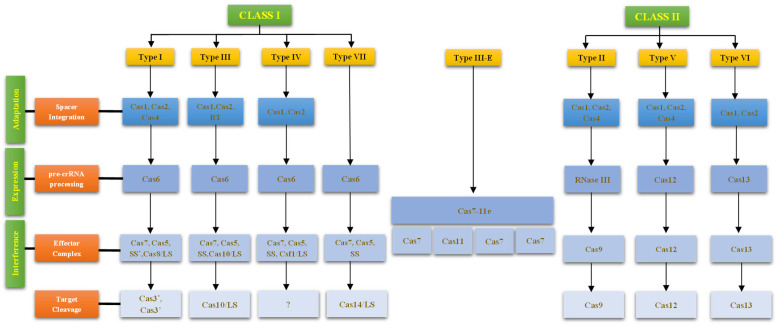
Functional diversity of CRISPR-associated proteins. The schematic representation provides an overview of the CRISPR-Cas system classification and function. CRISPR-Cas systems are divided into Class 1 (Types I, III, IV, VII) with multi-subunit complexes and Class 2 (Types II, V, VI) with single effectors like Cas9, Cas12, and Cas13. Subtype III-E is unique within Class 1, featuring a single multidomain effector protein. Cas proteins function in adaptation, expression/processing, and interference, highlighting the diversity of prokaryotic immune mechanisms. In the figure, LS, SS, and RT denote the large subunit and small subunit of the multi-protein effector complexes and reverse transcriptase in Class 1 CRISPR systems. The symbol “?” indicates that the effector nuclease for Type IV systems remains unidentified or poorly understood, whereas “*” highlights key effector proteins involved in target cleavage during the interference stage.

Based on the structural organization of their effector complexes, CRISPR-Cas systems are broadly categorized into two major classes, Class 1 and Class 2 (Figure 1). Class 1 systems include Types I, III, IV, and VII, which account for approximately 90% of all CRISPR-Cas loci. These systems utilize multi-subunit effector complexes for target interference, such as the Cascade complex in Type I and Csm/Cmr complexes in Type III [33]. The effector complexes of the Type I Class 1 CRISPR-Cas system are composed of multiple Cas proteins that assemble into a ribonucleoprotein complex responsible for recognition and cleavage of target DNA through crRNA-guided base pairing. Following target recognition, the Cascade complex recruits the nuclease–helicase Cas3, which mediates the degradation of the invading DNA. These complexes typically contain a large subunit (LS) that provides structural stability and participates in target DNA interaction, and a small subunit (SS) that contributes to complex assembly and nucleic acid binding. The CRISPR-Csm complex from Type III prokaryotic immune systems enables targeted RNA degradation in both the nucleus and cytoplasm, providing a programmable platform for precise RNA manipulation. In contrast, precise transcript targeting using conventional non-CRISPR RNA-targeting approaches in mammalian cells is often limited by low efficiency and subcellular delivery barriers. Furthermore, unlike CRISPR-Cas13 systems, the Csm complex minimizes off-target trans-cleavage, offering improved specificity for RNA manipulation [34].

In contrast, Class 2 systems, which comprise about 10% of CRISPR-Cas loci, include Types II, V, and VI. These are characterized by a single, large effector protein, Cas9 in Type II, Cas12 in Type V, and Cas13 in Type VI, which are responsible for target recognition and cleavage [35]. Due to their simpler architecture and ease of programmability, Class 2 systems, particularly Cas9 and Cas12, have emerged as powerful tools for genome editing and therapeutic applications.

Within these two classes, CRISPR-Cas systems are further classified into seven major types (Types I–VII) and over 46 subtypes, each distinguished by specific signature Cas proteins and their target molecules. Type I systems (Class 1) are defined by the signature protein Cas3, a helicase–nuclease that degrades DNA in a processive manner and includes subtypes I-A to I-G. Type II systems (Class 2) utilize Cas9, which introduces site-specific DSBs in DNA via its RuvC nuclease and HNH domains. The development of CRISPR SWAPnDROP, a CRISPR-Cas9-mediated genome engineering platform, enables large, marker-free insertions (up to 150 kb) across diverse bacterial species, showcasing its potential for robust interspecies chassis engineering [36]. Type III systems (Class 1), marked by Cas10, are unique in their ability to target both DNA and RNA, indicating a more versatile antiviral defense [37,38]. Subtype III-E is unusual among Class 1 as it utilizes a single multidomain effector protein. Type IV systems (Class 1), characterized by the Cas8-like protein Csf1, are less well understood, with unclear target specificity and subtypes IV-A and IV-B [39]. Meanwhile, Type V and VI systems (Class 2) have garnered increasing interest for their novel functionalities. Cas12 (Type V) targets DNA like Cas9 but also exhibits collateral cleavage of single-stranded DNA (ssDNA), a feature used in diagnostics platforms such as DETECTR [40]. Cas13 (Type VI) is distinct in its exclusive RNA targeting capability and its collateral cleavage of non-target RNAs, enabling applications in RNA knockdown and the development of diagnostic tools like SHERLOCK [41]. The Type VII CRISPR-Cas system possesses a unique effector protein, Cas14, formed by a β-CASP RNase domain fused to a Cas10 C-terminal homologous domain [42]. The functional diversity and evolutionary specialization of CRISPR-Cas systems not only highlight their significance in microbial immunity but also underscore their transformative potential in biotechnology, medicine, and molecular diagnostics.

The CRISPR-Cas system demonstrates remarkable evolutionary plasticity, primarily driven by its ongoing co-evolution with mobile genetic elements such as phages, plasmids, and transposons. HGT plays a crucial role in disseminating CRISPR-Cas modules across diverse prokaryotic species and domains, contributing to their extensive variability [43]. A recent study reports that CRISPR-Cas systems are found in about 90% of archaea and 40% of bacteria, and highlights their dual role in defense and regulation of HGT [44]. Phylogenetic analysis indicate that Class 1 systems are evolutionarily more ancient, while Class 2 systems likely emerged later, potentially evolving from mobile genetic elements such as transposon-associated protein B (TnpB) transposases, which are believed to be the ancestral precursors of Cas12 [45]. The dynamic nature of CRISPR loci is further underscored by frequent events of gene loss, rearrangement, and recombination, often resulting in hybrid systems and orphan CRISPR loci (those that lack associated *Cas* genes). Additionally, the co-localization of *Cas* genes with other mobile genetic elements, including prophages and integrative conjugative elements, highlights the modular exchange and fluidity of these systems. Recent discoveries of miniature CRISPR effectors, such as Cas14 and CasΦ, further exemplify the evolutionary adaptability of CRISPR-Cas systems. These compact variants are especially promising for therapeutic genome editing, as their small size facilitates efficient packaging and delivery using vectors like AAVs [46,47]. A recent study by Gencay et al. describes phage-engineered CRISPR-Cas antimicrobial systems targeting biofilm-forming *E. coli*, adding evidence for precise, phage-based delivery approaches in microbes [48]. Recent studies have shown the development of CRISPR-Cas3-enhanced phage therapy to target antibiotic-resistant *E. coli* [49], illustrating real-world clinical applications of phage-delivered CRISPR systems. Cas14 (also known as Cas12f), a compact nuclease (400–700 amino acid residues), overcomes PAM constraints with minimal or no PAM requirement and has been biochemically confirmed to cleave double-stranded DNA (dsDNA). Its engineered variants, such as the compact CasMINI system, further enhance genome-editing potential by combining the small size with broad PAM flexibility, expanding the range of editable genomic targets [46].

Advances in metagenomic mining have played a pivotal role in the discovery and classification of novel CRISPR-Cas systems, significantly expanding the known diversity of Cas proteins. High-throughput, culture-independent analysis of metagenomic assemblies enables the identification of compact and previously uncharacterized Cas effectors from uncultivated microbial communities. By exploring diverse environments such as soil, marine ecosystems, and host-associated microbiomes, this approach has greatly broadened the CRISPR-based genome-editing toolbox. Metagenomic datasets are typically analyzed using in silico pipelines that detect CRISPR arrays and associated *Cas* genes from assembled contigs [50,51]. Hidden Markov Model (HMM)-based searches [52] and computational tools such as CRISPRCasFinder [53], MinCED [54], and HMMER [55] are commonly employed to identify CRISPR loci and candidate Cas proteins. Subsequent phylogenetic analysis using tools such as BLAST, HHpred, and Clustal Omega help classify newly discovered proteins and determine their evolutionary relationships [56,57]. Spacer–protospacer matching further facilitates the prediction of PAM sequences, which are critical for target recognition. Using these approaches, several compact CRISPR effectors, including CasX (Cas12e), CasY (Cas12d), and CasΦ, have been identified from metagenomic datasets, providing nucleases with smaller sizes, distinct PAM requirements, and improved targeting flexibility [58]. The phage-encoded CasΦ nuclease employs a single catalytic site to function as both gRNA maturation and DNA cleavage for nucleic acid detection and genome editing [47]. Functional validation of candidate systems identified through these computational pipelines is an essential step to confirm their genomic- and biochemical-editing potential. This is typically performed through genome-editing experiments and cell-free transcription–translation (TXTL) systems (e.g., *E. coli* TXTL). Additionally, genome-editing experiments in bacterial and mammalian cells are done to validate system functionality in living contexts. In bacterial systems, plasmid interference assays are commonly used to assess nuclease activity and PAM compatibility, while mammalian cell-based assays are used to evaluate PAM compatibility, target specificity, and editing efficiency, thereby bridging computational prediction with experimentally validated function [59,60,61]. These discoveries not only expand the CRISPR toolkit but also contribute to the continuous refinement of CRISPR-Cas classification frameworks.

The diversity and classification of CRISPR-Cas systems reflect a remarkable example of microbial innovation in adaptive immunity. Advances in metagenomic mining and machine learning-based bioinformatics tools continue to reveal new subtypes and variants, expanding the CRISPR toolkit for applications ranging from genome editing to diagnostics and synthetic biology.

## 3. Genomic Landscape and Organization

**Figure 2:**
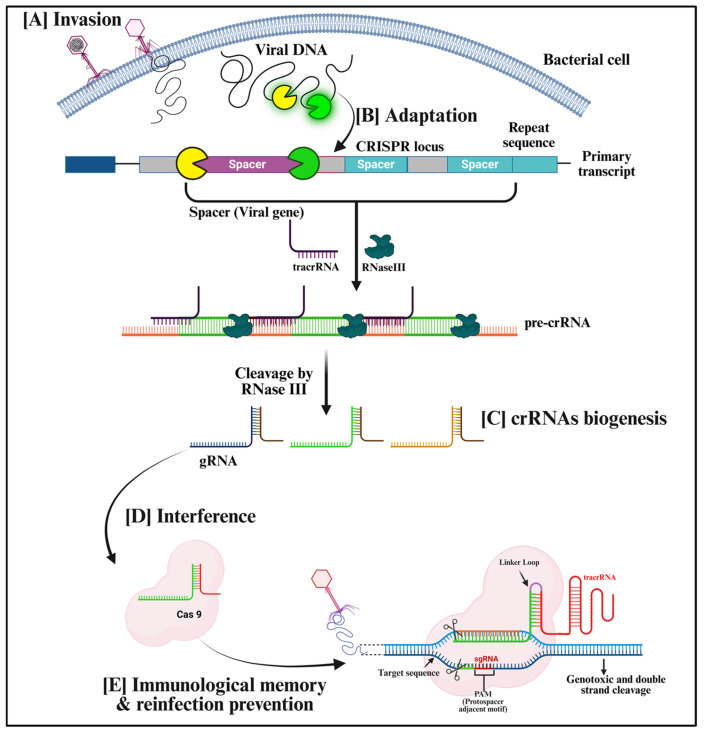
Diagrammatic illustration of stages of CRISPR-Cas adaptive immunity. The schematic representation depicts the three key phases of the CRISPR-Cas immune response in prokaryotes following the (**A**) invasion of viral DNA into a bacterial cell. (**B**) Adaptation phase, in which the foreign DNA (e.g., from a phage) is recognized, and the Cas1–Cas2 complex excises a protospacer sequence, integrating it into the host CRISPR array near the leader region. (**C**) Biogenesis of CRISPR-RNAs (crRNAs), in which the CRISPR array is transcribed into a precursor crRNA (pre-crRNA), which is then processed into individual crRNAs containing a unique spacer and partial repeat sequence. In the (**D**) interference phase, Cas effector proteins (or Cascade complexes) bind these crRNAs to form surveillance complexes that recognize and bind complementary sequences in invading genetic elements, leading to target cleavage and degradation. (**E**) Immunological memory and reinfection prevention: If the same virus infects the bacterium again, the previously generated crRNA–Cas9 complex rapidly recognizes PAM-associated target sequences in the viral genome and cleaves the DNA, thereby blocking reinfection. The conceptual framework is adapted from “Strategies to overcome the main challenges of the use of CRISPR/Cas9 as a replacement for cancer therapy” [69].

The CRISPR-Cas system is genomically organized into two main functional components, the CRISPR array and the *Cas* gene operon, typically found in near proximity within the host genome. The CRISPR array comprises short repetitive DNA sequences (known as repeats) interspersed with unique sequences (spacers) derived from previously encountered foreign genetic elements such as viruses or plasmids. These spacers serve as a molecular memory, allowing the host to recognize and defend against subsequent invasions. The length, sequence composition, and number of repeats and spacers vary considerably across species and even among strains, reflecting the adaptive and dynamic nature of CRISPR loci [62]. Upstream or adjacent to the CRISPR array lies the *Cas* gene cluster, which encodes proteins responsible for the three fundamental stages of CRISPR-mediated immunity: adaptation, expression, and interference (Figure 2). Adaptation, also referred to as spacer acquisition or insertion, is the initial phase where foreign DNA fragments are integrated into the CRISPR array of the host genome. This integration is facilitated by the conserved Cas1–Cas2 protein complex, composed of four Cas1 and two Cas2 subunits, which directs spacer integration at the leader sequence of the CRISPR locus, a short AT-rich regulatory region located upstream of the CRISPR array that guides the insertion of newly acquired spacers. Distinct mechanisms of spacer integration have been described for different CRISPR types. In Type I systems, the integration host factor (IHF) binds to the leader sequence and induces a structural bend in the DNA that enables the Cas1–Cas2 complex to initiate spacer insertion. Conversely, in the type II system, the Cas1 subunit specifically recognizes a region known as the leader anchoring sequence (LAS), guiding the directional integration of the new spacer [63]. Recent studies demonstrate that spacer acquisition is a highly conserved yet mechanistically diverse process centered around the Cas1–Cas2 integrase complex, but with substantial variation across various types and subtypes of CRISPR-Cas. These include differences in accessory proteins (e.g., Cas4, Cas3, Csn2), sources of prespacers (RecBCD/AddAB pathways), and modes of acquisition such as naïve and primed adaptation, rather than a strict classification based on Type I versus Type II systems [64]. Moreover, emerging evidence from Type III and Type V systems highlights that spacer acquisition mechanisms can be independent of interference modules and may involve distinct or minimal protein requirements, further underscoring the diversity of adaptation pathways [65]. Recent work also emphasizes the dynamic regulation of spacer acquisition and the involvement of additional factors such as Cas9 in Type II systems [66]. The second phase, expression, also known as crRNA biogenesis or processing, involves the transcription of the CRISPR array into a long precursor RNA, known as pre-crRNA, which is subsequently processed into smaller mature crRNAs. This processing occurs through cleavage within repeat sequences, followed by additional trimming at the 5′ or 3′ ends. The enzymes responsible for crRNA maturation vary among different CRISPR-Cas types and subtypes. In the final interference stage, mature crRNAs assemble with Cas proteins to form effector complexes that recognize and cleave complementary sequences in invading nucleic acids [67]. Target recognition in many systems additionally requires the presence of a PAM, a short conserved sequence in the target DNA that enables discrimination between foreign DNA and the host genome, thereby preventing self-targeting and ensuring accurate cleavage [68].

The organization and composition of the Cas operon vary among different CRISPR-Cas types and subtypes, comprising distinct sets of core and accessory genes, such as *Cas1* and *Cas2*, which are highly conserved across almost all CRISPR-Cas systems and are primarily involved in spacer acquisition (adaptation). In contrast, type-specific genes encode effector proteins (e.g., Cas9, Cas12, and Cas13) or components of multi-protein complexes such as Cascade in Type I systems and Csm/Cmr complexes in Type III systems [62]. CRISPR-Cas loci are frequently located near mobile genetic elements such as transposons, integrative conjugative elements, or prophages. This genomic proximity reflects the evolutionary history of these systems, which has been shaped by HGT and modular genetic rearrangements. Structural organization also differs between the two major CRISPR classes. Class 2 systems typically exhibit compact architectures in which a single multidomain effector protein mediates target recognition and cleavage, whereas Class 1 systems possess more complex operons encoding multiple effector subunits [62]. CASTs are hybrid molecular systems that combine RNA-guided CRISPR targeting with Tn7-like transposition machinery. Structurally, CASTs typically consist of a CRISPR effector module, such as Cascade or Cas12k, which is genomically co-localized with transposition genes, including tnsB, tnsC, and tniQ (or tnsD), forming a compact operon-like structure. This arrangement enables coordinated expression and functional coupling between the targeting and integration components. The CRISPR effector complex associates with a gRNA to direct sequence-specific DNA recognition, subsequently recruiting the transposase machinery, which mediates the integration of donor DNA into the target genomic locus. Unlike conventional CRISPR-based editing systems, CASTs enable RNA-guided insertion of large DNA fragments without generating DSBs, providing a reliable platform for programmable genome engineering [70,71]. Additionally, genomic variations have revealed the presence of orphan CRISPR arrays, which lack adjacent *Cas* genes, and degenerate CRISPR systems containing non-functional Cas remnants. These configurations likely represent evolutionary relics or domesticated elements derived from ancestral defense systems [72].

The CRISPR array transcription is mainly controlled by a promoter located upstream of the first repeat, producing a long pre-crRNA that is later cleaved into individual crRNAs, each containing a spacer-repeat unit. The orientation, strand specificity, and regulatory elements within the CRISPR-Cas loci are critical for efficient CRISPR function and are tightly regulated by both host and environmental factors. Collectively, the genomic landscape of CRISPR-Cas systems represents a highly diverse and modular genetic architecture that underpins their versatility in microbial adaptive immunity and their expanding applications in genome engineering.

## 4. Biotechnological Applications of CRISPR-Cas Systems in Microorganisms

CRISPR-Cas systems have emerged as powerful tools for engineering industrial microorganisms, offering high precision, efficiency, and programmability in genome editing. Industrial chassis microbes such as *E. coli*, *Corynebacterium glutamicum*, *Bacillus subtilis*, and *Saccharomyces cerevisiae* serve as foundational platforms for producing pharmaceuticals, nutraceuticals, chemicals, enzymes, and fuels. Through targeted genetic modification and metabolic pathway optimization, these microbes can be transformed into efficient microbial cell factories for large-scale bioproduction [73]. A summary of major CRISPR-Cas-based therapeutic strategies and their outcomes is presented in Table 2.

### 4.1. Multiplexed Gene Knockouts/Knock-Ins for Pathway Construction

Multiplexed CRISPR-Cas technologies have enabled precise genome and metabolic engineering by allowing the simultaneous editing of multiple genetic loci for pathway construction. This enables the coordinated gene knockouts of competing pathways alongside knock-ins of productive or heterologous genes within a single step. This multiplexing strategy allows the concurrent use of multiple gRNAs. The rewiring of cellular metabolic networks in a coordinated manner facilitates the redirection of metabolic flux by eliminating competing pathways and introducing biosynthetic genes. This redirects the carbon flux toward desired biosynthetic outputs, thereby accelerating the development of industrially relevant microbial strains [82].

Cas9- and Cas12a-based systems are widely used for multiplex editing, with Cas12a offering an advantage due to its ability to process crRNA arrays for simultaneous targeting. These approaches have been successfully applied in microbial chassis to enhance the production of amino acids, biofuels, and organic acids as well as to construct and optimize complex metabolic pathways. For instance, in *Saccharomyces cerevisiae*, simultaneous disruption of multiple genes has resulted in a dramatic increase in mevalonate production, a key precursor for isoprenoids [83]. Similarly, extensive multiplex editing in *E. coli* has enabled improved β-carotene production [84]. In more complex pathway reconstruction, simultaneous knockout of competing reactions combined with the knock-in of multiple heterologous genes has enabled the biosynthesis of non-native compounds such as 1,4-butanediol (1,4-BDO) [85]. Beyond microbes, multiplexed CRISPR systems have also been applied in plants, where targeting multiple genes within a biosynthetic pathway (e.g., the GABA shunt in tomato) has yielded substantial metabolite accumulation, demonstrating the versatility of this approach across biological systems [86].

Mechanistically, multiplexed editing is facilitated by advanced strategies such as gRNA arrays, where multiple gRNAs are expressed as a single transcript and subsequently processed into individual functional units via endogenous tRNA-processing systems or engineered nucleases like Csy4 [87]. Furthermore, integration of multiplexed DSBs with homology-directed repair (HDR) enables the precise insertion of multiple genes across different genomic loci, while base editing technologies provide an alternative route for introducing multiple point mutations with reduced cytotoxicity [88].

Overall, multiplexed CRISPR-based knockout and knock-in strategies offer significant advantages, including marker-free genome engineering, high-throughput combinatorial modifications, and efficient manipulation of polygenic traits. This approach is most suitable when permanent and large-scale rewiring of metabolic networks is required, particularly for constructing non-native biosynthetic pathways or eliminating competing flux routes. It is therefore preferred in industrial strain development where stable, heritable genetic modifications are essential. However, challenges such as increased cytotoxicity due to simultaneous DSBs, potential chromosomal rearrangements, and competition among multiple gRNAs for Cas proteins can limit editing efficiency. Despite these constraints, continued advancements in CRISPR system design and delivery are expected to further enhance the robustness and scalability of multiplexed pathway engineering.

### 4.2. CRISPR-Based Dynamic Transcriptional Regulation

**Figure 3:**
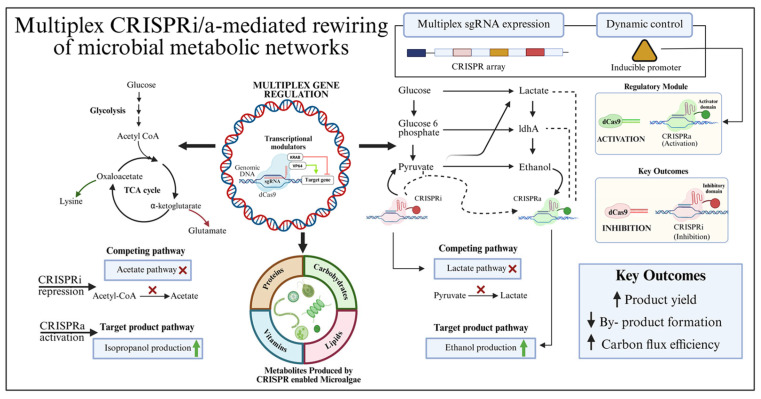
Multiplex CRISPRi/a-mediated rewiring of microbial metabolic networks. Schematic illustration of multiplexed CRISPR interference (CRISPRi) and CRISPR activation (CRISPRa) systems enabling simultaneous repression and activation of multiple genes to redirect metabolic flux. A programmable CRISPR array expressing multiple sgRNAs under an inducible promoter facilitates dynamic and coordinated gene regulation. CRISPRi (generally uses dCas9 guided by sgRNA in bacterial systems, whereas in eukaryotic systems it is often fused to repressor domains) inhibits competing metabolic pathways such as acetate and lactate formation, while CRISPRa (dCas9 fused to activator domains) enhances target product pathways, including isopropanol and ethanol biosynthesis. Central carbon metabolism (glycolysis and TCA cycle) is strategically rewired to increase flux toward desired products while minimizing by-product formation. Additionally, multiplex gene regulation via transcriptional modulators enhances metabolite production in CRISPR-enabled microalgae. Overall, this multiplexed regulatory strategy improves product yield, reduces metabolic burden from competing pathways, and enhances carbon flux efficiency. The “X” symbol denotes the inhibition step.

CRISPR-based transcriptional regulation technologies, such as CRISPRi and CRISPRa, enable reversible and tunable control of gene expression using catalytically inactive Cas proteins such as dCas9. Mechanistically, CRISPRi employs dCas proteins guided by sgRNAs to sterically block RNA polymerase binding or elongation, resulting in transcriptional repression, whereas CRISPRa recruits transcriptional activators to enhance gene expression. These systems allow precise control of gene expression without altering genomic sequences, making them particularly suitable for fine-tuning metabolic pathways. CRISPRi/a are widely used to rebalance metabolic flux by repressing competing pathways or activating rate-limiting steps (Figure 3) [89]. For instance, modular and signal-responsive genetic integrating biosensor inputs with the FnCas12a system enable programmable, inducible control of gene expression in *E.coli*, facilitating dynamic metabolic reprogramming [90]. Similarly, in *Bacillus licheniformis*, multiplex CRISPRi-mediated repression of by-product formation and L-valine degradation pathways significantly enhances L-valine production, particularly via combinatorial targeting of *alsD* and *bcd* [91]. In *Bacillus subtilis*, a xylose-inducible CRISPRi system enables temporal regulation of key metabolic genes, allowing coordinated glucose–xylose utilization and optimized N-acetylglucosamine production [92].

Despite these advances, the efficiency of CRISPRi/a is influenced by factors such as gRNA design, target site accessibility, chromatin or DNA context, and host-specific transcriptional machinery. Off-target effects, leaky repression, and challenges in delivery and expression of CRISPR components may further limit performance. CRISPRi/a is particularly advantageous when reversible, tunable, and condition-dependent control of gene expression is required, without altering the genomic sequence. It is ideal for fine-tuning metabolic flux and balancing pathway intermediates in systems where static modifications may lead to metabolic burden or toxicity. Additionally, achieving optimal dynamic regulation often requires iterative circuit tuning and careful balancing of gene expression levels. Nonetheless, CRISPRi/a remains a powerful and versatile tool for dynamic, systems-level metabolic engineering and gene regulation.

### 4.3. Biosensor Integration and CRISPR-Guided Directed Evolution

CRISPR technologies are increasingly integrated with biosensors and directed evolution strategies to accelerate microbial strain optimization. CRISPR-based biosensors enable real-time detection of intracellular metabolites, environmental signals, or genetic changes, facilitating high-throughput and precise screening. This transformative approach in synthetic biology effectively bridges the gap between high-precision genetic engineering and high-throughput screening [93,94]. Specifically, CRISPR-mediated genome editing allows targeted genetic modifications but is typically limited in throughput, whereas conventional high-throughput screening methods can process large mutant libraries but often lack direct and precise linkage between phenotype and genotype. Integration of biosensors with CRISPR systems overcomes the limitation through the direct coupling of targeted mutagenesis with real-time, phenotype-linked signal outputs. This strategy creates a powerful feedback loop in which CRISPR systems (e.g., EvolvR, CasPER) introduce targeted genetic diversity, while genetically encoded biosensors convert phenotypic outputs such as metabolite levels into measurable signals like fluorescence or antibiotic resistance. This approach enables rapid identification of rare, high-performing variants from large mutant libraries, overcoming key bottlenecks in metabolic engineering and enzyme optimization. In advanced applications, biosensors can dynamically regulate CRISPR activity, triggering targeted mutagenesis only under suboptimal production conditions, thereby driving adaptive evolution toward enhanced productivity [95,96].

The practical application of this integration is particularly evident in the development of smart microbial factories and advanced diagnostics. For instance, biosensors can be engineered to trigger the CRISPR-guided mutation of specific enzymes only when product levels are low, effectively forcing the cell to evolve toward higher productivity [97]. Coupling CRISPR-mediated mutagenesis with biosensor-guided screening significantly accelerates directed evolution compared to conventional random mutagenesis approaches, enabling efficient identification of strains with enhanced metabolite production, stress tolerance, or novel functionalities. Beyond strain engineering, CRISPR-based biosensing platforms such as SHERLOCK (Cas13-based) and DETECTR (Cas12-based) have revolutionized molecular diagnostics by enabling rapid, sensitive, and specific detection of nucleic acids. These systems are widely applied for detecting viral and bacterial pathogens, including COVID-19, as well as environmental toxins and foodborne contaminants [98]. Additionally, CRISPR-engineered microbial biosensors have been developed for monitoring heavy metals such as cadmium and lead, further expanding their environmental and industrial applications [99]. Specific high-sensitivity enzymatic reporter unlocking (SHERLOCK) uses Cas13 to target RNA, enabling the detection of viral pathogens. DNA endonuclease-targeted CRISPR trans-reporter (DETECTR) utilizes Cas12 to target DNA, allowing rapid, visual detection of bacteria and viruses [100]. Cas12/13 systems are used to monitor contaminants, including toxins from harmful algal blooms and foodborne pathogens like *Salmonella* and *E. coli*. Label-free and visual detection methods using Cas12a-based biosensors and syringe filters are being developed for food safety monitoring [101].

This strategy is best applied when pathway behavior is complex or poorly understood, enabling adaptive optimization through iterative mutation and selection. It is especially valuable for evolving high-performing strains or enzymes where rational design alone is insufficient. However, challenges remain, including the design of robust and specific biosensor circuits, minimization of background noise and false signals, and the scalability of high-throughput screening platforms. Despite these limitations, the integration of CRISPR-guided evolution with biosensing represents a transformative strategy for next-generation synthetic biology, with broad applications in sustainable bioproduction, environmental monitoring, and precision diagnostics.

## 5. Challenges and Limitations

Despite its transformative impact on microbial biotechnology, CRISPR-based research faces several technical and ethical challenges that must be addressed for broader and safer applications.

### 5.1. Specificity Issues and Off-Site Mutations

A key concern in CRISPR applications is the potential for off-target activity, in which the Cas nuclease, guided by sgRNA, binds and cleaves unintended genomic sites that are partially complementary to the intended target sequence. Such off-target activity can lead to undesired mutations, insertions, or other deletions at non-target sites, potentially disrupting essential genes, genome stability, or regulatory elements [102]. In microbial systems engineered for applications such as biofuel production, bioremediation, pharmaceutical synthesis, or therapeutic delivery, off-target effects of CRISPR-Cas systems pose significant concerns. Unintended genomic edits can disrupt essential metabolic pathways, reduce production yields, or lead to unpredictable phenotypic changes, particularly in industrially important or probiotic strains. These off-target effects primarily result from the mismatch tolerance of Cas nucleases, especially in PAM-distal regions of the target site. SpCas9, widely used in microbial genome engineering, is known for its susceptibility to off-target cleavage in regions of sequence similarity [103]. Recent advances have introduced several precision-enhancing strategies to address this limitation. High-fidelity Cas9 variants, such as eSpCas9 and SpCas9-HF1, have been designed to minimize off-target activity without compromising on-target precision [104]. However, these variants may sometimes exhibit reduced editing efficiency in certain microbial hosts, highlighting the need for careful optimization.

Another promising solution is the use of PEs and BEs, which enable targeted nucleotide substitutions or small insertions without generating DNA DSBs. These approaches substantially reduce the unintended genomic rearrangements and have shown improved precision in microbial genome engineering. Additionally, the fusion of additional functional domains to PEs has been explored to further enhance editing efficiency. Nevertheless, their editing scope remains limited by sequence context and editing window constraints [105].

Additionally, the use of truncated gRNAs (17–18 nucleotide) mitigates nonspecific binding by lowering affinity at mismatched sites, thereby enhancing targeting specificity [106]. Recent developments in deep learning- and machine learning-based gRNA design models further enhance CRISPR specificity. These computational models analyze sequence features, chromatin accessibility, and mismatch tolerance patterns to predict both editing efficiency and off-target risk before experimental implementation [107,108]. These advancements are important for ensuring the precision and reliability of CRISPR-based microbial engineering.

### 5.2. Overcoming Delivery Inefficiencies in Diverse Microbial Hosts

Efficient delivery of CRISPR components, including Cas proteins and gRNAs, remains a major technical challenge, particularly in non-model microorganisms, extremophiles, and industrial microbial strains. Conventional delivery approaches such as electroporation, chemical transformation, or plasmid conjugation are often strain-specific and may be inefficient due to factors such as restrictive cell walls, endogenous restriction-modification systems, or incompatibility with host expression machinery. Designing host-specific expression systems for CRISPR components is often labor-intensive and may not always yield efficient editing outcomes due to differences in promoter compatibility and intracellular conditions. To overcome this barrier, innovative approaches such as light-activated gRNA systems based on photocatalytic chemistry have recently been developed. In this strategy, modification of vinyl ether triggered by visible light enables precise regulation of guide RNA activity, functioning as a controllable CRISPR “ON/OFF” switch. This approach allows temporal control of Cas9 activity and can help reduce unintended editing events, although further optimization is required for broader microbial applications [109].

Recent studies have explored bacteriophage-mediated delivery systems, which have emerged as an effective strategy for introducing CRISPR components into bacteria with high specificity. Engineered bacteriophages can deliver CRISPR-Cas payloads directly into target bacterial populations, enabling both genome editing and selective elimination of specific bacterial strains. For example, *Clostridioides difficile*, a temperate bacteriophage engineered to deliver the CRISPR-Cas3 system, enables targeted bacterial clearance through Cas3-mediated nuclease activity [110]. However, the narrow host range of bacteriophages and potential resistance mechanisms in bacterial communities remain important limitations.

Another promising approach involves engineered conjugative CRISPR plasmids, which differ from conventional plasmid conjugation by enabling the targeted and efficient transfer of genome-editing constructs between bacterial cells. Unlike standard conjugation methods that primarily serve as a general DNA delivery mechanism and may suffer from host-specific limitations, these systems are designed to disseminate CRISPR components in a sequence-specific manner. Importantly, conjugation-based delivery demonstrates relatively high transfer efficiencies in many Gram-negative bacteria and moderate efficiencies in Gram-positive systems, with minimal cytotoxicity compared to physical or chemical transformation methods. Additionally, these systems can accommodate large DNA cargo, including entire CRISPR-Cas operons and donor templates, making them particularly suitable for complex genome engineering. Due to their scalability, low toxicity, and compatibility with diverse microbial communities, conjugative systems are currently among the most practical approaches for industrial and microbiome-level applications [111,112]. This strategy has been widely applied in enterobacterales to target antibiotic-resistance genes such as β-lactamase (*bla*) and colistin-resistance (*mcr-1*), enabling the resensitization of resistant strains to antimicrobials [113]. Conjugative systems have also been used to target resistance genes in *Enterococcus faecalis* within gut microbial communities [114].

Emerging nanoparticle-based delivery mechanisms, including polymer-based nanocarriers and lipid nanoparticles, are also being examined for transporting CRISPR ribonucleoprotein complexes into microbial cells. These systems offer the advantage of transient DNA-free editing without stable integration of plasmid, thereby minimizing the risk of genomic alterations [115]. However, their application in microbes remains limited by relatively low transformation efficiencies, particularly due to barriers such as rigid bacterial cell walls. In addition, certain nanomaterials may exhibit dose-dependent cytotoxicity and membrane disruption, which can compromise cell viability. Cargo size is also constrained, as nanoparticles are generally optimized for smaller CRISPR ribonucleoprotein complexes rather than large DNA constructs [116]. While approaches such as cell nanoporation enable exosomes to function as universal nucleic acid carriers, enhancing Cas9 mRNA loading and transfer efficiency, these systems are still in the early stages of development and lack scalability for routine industrial use [117,118]. The literature studies have substantiated the use of polymeric nanoparticles for facilitating CRISPR-Cas ribonucleoprotein complexes into bacterial cells [119]. Phage-based delivery systems provide another alternative by exploiting natural infection mechanisms for efficient DNA transfer. These systems can achieve high delivery efficiency and species specificity, particularly in targeting pathogenic bacteria. However, their utility is often restricted by limited cargo capacity, host range specificity, and potential immune or resistance responses. Furthermore, large-scale production and standardization of engineered phages remain challenging [120,121]. Collectively, while nanoparticle- and phage-based approaches offer innovative and potentially precise delivery strategies, conjugative plasmid systems currently remain the most effective and scalable method for microbial genome engineering, particularly in industrial and environmental applications, due to their higher efficiency, lower toxicity, and ability to deliver large genetic payloads. CRISPR applications in plants face unique challenges due to large, polyploid genomes, high repetitive content, and rigid cell walls that limit efficient delivery and increase off-target risks. To overcome these barriers, strategies such as nanoparticle and viral-mediated delivery, in planta transformation, base and prime editors, optimized guide design, PAM-flexible Cas variants, and transient expression systems are enabling precise, heritable plant genome edits for enhanced stress tolerance, nutrient efficiency, and disease resistance [122].

In addition, cell-free CRISPR systems integrated with microfluidics-assisted in vitro compartmentalization offer an effective alternative to overcome intracellular delivery barriers. These platforms enable high-throughput screening of CRISPR-Cas activity in double emulsion droplets, linking genotype to phenotype via compartmentalized gene amplification and reporter-based detection [123]. Collectively, these emerging technologies represent significant advances toward overcoming CRISPR delivery barriers.

### 5.3. Ethical, Biosafety, and Regulatory Barriers

The application of CRISPR-edited microorganisms also raises significant biosafety, ethical, and regulatory challenges, particularly when genetically modified microorganisms (GMMs) are intended for environmental, industrial, or therapeutic use. One of the major concerns is the potential accidental release of engineered microbes, which could disrupt natural ecosystems or facilitate the horizontal transfer of engineered genes, including antimicrobial resistance determinants. To mitigate these risks, several biocontainment strategies are being developed. Synthetic biology approaches such as genetic kill switches, auxotrophic dependency systems, and programmable gene circuits allow engineered microbes to survive only under specific laboratory conditions [124,125]. These strategies significantly reduce the risk of uncontrolled environmental proliferation but may impose metabolic burdens that affect microbial productivity.

The safe use of CRISPR-edited microorganisms faces several critical regulatory and governance challenges. Many existing regulatory frameworks are fragmented and lag behind the rapid advancements in genome editing and synthetic biology. Regulatory approaches such as the European Union follow a process-based framework, classifying organisms as GMMs based on the techniques employed, regardless of the presence of foreign DNA. In contrast, countries such as India, the US, and Argentina adopt product-based regulatory approaches, focusing on the characteristics of the final organism, where certain genome edits may be subject to reduced regulatory oversight. While international agreements such as the Cartagena Protocol on Biosafety and the Biological Weapons Convention provide foundational guidance on biosafety and biosecurity, they are not fully equipped to address the complexities introduced by modern genome-editing technologies [126,127]. Despite its advancements, CRISPR-based microbial engineering presents significant dual-use risks. CRISPR may enhance antimicrobial resistance, transmissibility, and pathogen virulence, or facilitate the synthesis of novel or extinct pathogens, raising biosecurity concerns. Increased accessibility further heightens misuse risks, while unintended HGT may drive ecological disruption. These challenges necessitate standardized biosafety governance, responsible research and innovation (RRI), and integrated dual-use risk assessment [128,129]. Environmental release of engineered microbes may disrupt soil ecosystems and human microbiota. Oversight remains constrained by gaps in frameworks such as the Biological and Toxin Weapons Convention, which lack strong verification and monitoring mechanisms. Moreover, despite its precision, CRISPR result in unpredictable outcomes due to unintended genomic changes that complicate long-term safety and risk assessment [130,131].

Addressing these challenges requires continued advancement in high-fidelity Cas variants, efficient delivery platforms, host-optimized expression systems, and robust ethical guidelines to ensure responsible and safer utilization of CRISPR technologies in microbial research. Therefore, it is essential to maintain equilibrium between safety, innovation, public trust and sustainability for the development of globally coordinated, integrated and flexible biosafety and regulatory frameworks.

## 6. Future Perspectives

The next generation of CRISPR technologies is rapidly surpassing conventional genome editing. Emerging platforms such as prime editing, base editing, and CASTs are redefining the scope of genetic manipulation, thereby improving editing fidelity and expanding applicability. In addition, recently identified compact and PAM-flexible nucleases, including Cas12, Cas13, and CasΦ, offer improved delivery potential and broader targeting range, making them highly suitable for non-model and industrially relevant microbial systems [12,132].

A key emerging area is the advancement of in situ and microbial–genome-editing technologies. Recent progress in phage-mediated delivery, extracellular vesicle-based approaches, and conjugative elements, combined with CRISPR-based antimicrobials and gene regulators, enables precise microbiome engineering in human health, agriculture, and environmental ecosystems [133].

Another transformative direction is the integration of CRISPR with automated bioengineering platforms and artificial intelligence (AI). Deep learning models and AI-driven tools are increasingly being employed for gRNA optimization, prediction of editing outcomes, and discovery of novel Cas variants with enhanced specificity and reduced immunogenicity. Coupled with high-throughput CRISPR screening and robotic automation, these systems enable scalable, data-driven optimization of metabolic pathways and genetic circuits, significantly accelerating synthetic biology and industrial biotechnology applications [134,135].

Emerging CRISPR-based approaches, such as CRISPR-based biosensors and diagnostic platforms (e.g., SHERLOCK and DETECTR), are rapidly expanding the functional landscape of CRISPR beyond genome editing. These technologies enable real-time detection of biomarkers and pathogens at low concentrations by exploiting the collateral cleavage activities of Cas effectors, enabling precision medicine, epidemiological tracking, and environmental surveillance [136].

Prospectively, CRISPR-based innovations in sustainable biotechnology play a significant role in addressing global challenges. Microbial engineering systems for plastic biodegradation, carbon sequestration, bioenergy production, and eco-friendly chemical synthesis represent an advancing era of research [137]. For instance, the overexpression of dioxygenase genes, or integration of synthetic catabolic modules, can enable microbes to break down complex pollutants like polyaromatic hydrocarbons (PAHs), polychlorinated biphenyls (PCBs), and microplastics more efficiently [138].

Furthermore, these advancements highlight a transition in CRISPR technologies from isolated genome-editing tools to programmable, integrated biological platforms. The convergence of CRISPR with multi-omics, systems biology, and metabolic modeling will facilitate the design of robust microbial cell factories with enhanced efficiency and environmental compatibility. The most significant progress is anticipated from the integration of AI-driven CRISPR design and in situ microbiome engineering that enables predictive and precise genome editing within complex biological environments. Integration of biotechnological strategies and biosensing approaches is expected to transform CRISPR applications across industrial biotechnology, healthcare and environmental remediation.

## 7. Conclusions

Conclusively, the CRISPR-Cas system is at the forefront of a new era in genetic engineering, offering powerful, versatile, and precise genome-editing capabilities in microbial systems. Its expanding toolbox, including advanced editors, novel Cas variants, and improved delivery strategies continues to broaden its applicability beyond traditional boundaries. The future of CRISPR lies in enabling precise manipulation of non-model organisms, enabling fine-scale manipulation of microbiomes, integrating with AI-driven design frameworks, and addressing global sustainability challenges through environmental applications. Continued innovation in CRISPR technology, delivery mechanisms, and computational tools, along with ethical oversight, will determine how profoundly this transformative tool reshapes science, medicine, agriculture, and industry.

Importantly, as CRISPR technologies continue to evolve, careful consideration of ethical, biosafety, and regulatory aspects will be essential to ensure responsible implementation. Overall, the ongoing expansion and refinement of the CRISPR toolkit positions it as a cornerstone technology with far-reaching implications for science, medicine, agriculture, and industry.

## Figures and Tables

**Table 2:** Overview of CRISPR-Cas-based biotechnological and therapeutic strategies and their outcomes.

S. No.	CRISPR-Cas-Mediated Approach	System Components	CRISPR Application	Outcomes	References
1.	Prime editing	Catalytically impaired Cas9 nickase (nCas9) fused to an engineered RT and guided by a pegRNA	Enables precise genome editing including insertions, deletions, and point mutations facilitating correction of disease-causing mutations such as sickle cell disease and Tay–Sachs disease and functional genomics studies	Precise and accurate editing in human cells with minimal by-products and lower off-target effects	[27]
2.	Single-homology-arm CRISPR-associated transposase (ShCAST) system	Type V-K CRISPR effector Cas12k, gRNA, and donor DNA cargo	RNA-guided site-specific DNA insertion for genome engineering in bacteria and microbial strain development for synthetic biology and industrial biotechnology	Efficient and programmable insertion of DNA segments downstream of the protospacer	[30]
3.	CRISPR-based cytosine base editing system	Nickase *Neisseria meningitidis* type II Cas 9 (nNme2Cas9) fused with engineered cytidine deaminase	Precise C→T base editing with expanded PAM compatibility for therapeutic correction and functional genomics, including gene validation and engineering in model organisms	Enable precise and efficient editing of genome in human cells and rabbit embryos with high specificity and minimal off-target effects	[74]
4.	CRISPR-Cas9-based antiviral strategy using Herpes simplex virus type 1 (HSV-1)-erasing lentiviral particles (HELPs)	SpCas9 mRNA and viral gene-targeting gRNAs delivered via mRNA-carrying lentiviral particles	Targeted cleavage of HSV-1 genomes to inhibit viral replication and development of CRISPR-based antiviral platforms	Efficiently blocked replication of HSV-1	[75]
5.	CRISPR-Cas9 edited T cell therapy	CRISPR-Cas9 with sgRNAs	Deletion of endogenous T cell receptor alpha (TCRα) and beta (TCRβ) chains to enable engineered T cells for adoptive cancer immunotherapy and immune cell reprogramming for translational and cellular biotechnology	Engineered T cells showed successful engraftment with multiplex CRISPR edits at multiple genomic sites	[76]
6.	Ex vivo CRISPR-Cas9 editing of programmed cell death protein-1 (PD-1) in T cells	Cas9 and sgRNA plasmids targeting the PD-1 gene	Immune checkpoint disruption for cancer therapy and immunomodulation studies in cell-based research	Edited T cells were successfully detected after infusion, with minimal off-target effects	[77]
7.	CRISPR-based precise genome editing	CRISPR-Cas systems with sgRNA targeting γ-globin (HBG) repressor genes (e.g., BCL11A)	Reactivation of fetal hemoglobin for hemoglobinopathy treatment and gene regulation studies in hematopoiesis	Increased HbF levels and improved disease phenotype	[78]
8.	CRISPR-Cas9 engineered chimeric antigen receptor (CAR) T cells therapy	Integration of CAR T cells and CRISPR-Cas9 gene-editing system	Precision engineering of CAR-T cells for cancer therapy and advancement of synthetic immunology and cell engineering platforms	Enhanced efficacy with reduced toxicities, including a lower risk of immune-related neurotoxicity (ICANS) and cytokine release syndrome (CRS)	[79]
9.	In vivo genome editing	Lipid nanoparticle (LNP) delivery system comprising Cas9 mRNA and sgRNA	Targeted gene editing in hepatocytes for ATTR treatment and development of in vivo gene delivery platforms	Reduction in serum TTR levels in dose-dependent manner	[80]
10.	Ex vivo genome editing targeting CCR5	CRISPR-Cas9 ribonucleoprotein (RNP) complexes targeting CCR5	Disruption of CCR5 for HIV therapy and gene-editing strategies in stem cell engineering	Successful engraftment of CRISPR-edited hematopoietic stem and progenitor cells (HSPCs); CCR5 disruption efficiency	[81]

## Data Availability

No new data were created or analyzed in this study. Data sharing is not applicable.

## Abbreviations

The following abbreviations are used in this manuscript:

AAV	Adeno-associated virus
AI	Artificial intelligence
ATTR	Transthyretin amyloidosis
BE	Base editor
CAR-T	Chimeric antigen receptor T cell
Cascade	CRISPR-associated complex for antiviral defense
CAST	CRISPR-associated transposase
Cas	CRISPR-associated protein
Cas10	CRISPR-associated protein 10
Cas12a	CRISPR-associated protein 12a
Cas13	CRISPR-associated protein 13
Cas3	CRISPR-associated protein 3
CasΦ	CRISPR-associated protein Phi
CasX	CRISPR-associated protein X (Cas12e)
CasY	CRISPR-associated protein Y (Cas12d)
CCR5	C-C chemokine receptor type 5
Cmr	CRISPR-associated complex subunit (Type III system)
CRISPR	Clustered regularly interspaced short palindromic repeat
CRISPRa	CRISPR activation
CRISPRi	CRISPR interference
CRS	Cytokine release syndrome
crRNA	CRISPR RNA
Csm	CRISPR-associated complex subunit (Type III system)
dCas9	Catalytically inactive Cas9
DETECTR	DNA endonuclease-targeted CRISPR trans reporter
DSB	Double-strand break
GMMs	Genetically modified microorganisms
gRNA	Guide RNA
HbF	Fetal hemoglobin
HBG	γ-globin
HELP	Herpes simplex virus type 1 erasing lentiviral particle
HGT	Horizontal gene transfer
HMM	Hidden Markov model
HSPCs	Hematopoietic stem and progenitor cells
HSK	Herpes simplex virus type 1-induced herpetic stromal keratitis
HSV-1	Herpes simplex virus type 1
ICANS	Immune effector cell-associated neurotoxicity syndrome
IHF	Integration host factor
LAS	Leader anchoring sequence
LNP	Lipid nanoparticle
LS	Large subunit
nCas9	Cas9 nickase
Nme2Cas9	*Neisseria meningitidis* type II Cas 9
nNme2Cas9	Nickase *Neisseria meningitidis* type II Cas 9
NSCLC	Non-small-cell lung cancer
PAH	Polycyclic aromatic hydrocarbon
PAM	Protospacer adjacent motif
PCB	Polychlorinated biphenyl
PD-1	Programmed cell death protein-1
PE	Prime editor
pegRNA	Prime editing guide RNA
pre-crRNA	Precursor-CRISPR RNA
RNP	Ribonucleoprotein
RT	Reverse transcriptase
sgRNA	Single-guide RNA
ShCAST	Single-homology-arm CRISPR-associated transposase
SHERLOCK	Specific high-sensitivity enzymatic reporter unlocking
SS	Small subunit
SpCas9	*Streptococcus pyogenes* Cas9
TCR	T cell receptor
TTR	Transthyretin
